# Physical, Behavioural and Genetic Predictors of Adult Hypertension: The Findings of the Kaunas Cardiovascular Risk Cohort Study

**DOI:** 10.1371/journal.pone.0109974

**Published:** 2014-10-14

**Authors:** Janina Petkeviciene, Jurate Klumbiene, Sandrita Simonyte, Indre Ceponiene, Kristina Jureniene, Vilma Kriaucioniene, Asta Raskiliene, Alina Smalinskiene, Vaiva Lesauskaite

**Affiliations:** 1 Faculty of Public Health, Medical Academy, Lithuanian University of Health Sciences, Kaunas, Lithuania; 2 Institute of Microbiology and Virology, Veterinary Academy, Lithuanian University of Health Sciences, Kaunas, Lithuania; 3 Department of Cardiology, Medical Academy, Lithuanian University of Health Sciences, Kaunas, Lithuania; 4 Institute of Cardiology, Medical Academy, Lithuanian University of Health Sciences, Kaunas, Lithuania; University of Texas Health Science Center at San Antonio, United States of America

## Abstract

**Background:**

The roots of adult hypertension go back to childhood. This study aimed to examine the independent effects of physical, behavioural and genetic factors identified in childhood and mid-adulthood for prediction of adult hypertension.

**Methods:**

The study subjects were participants of the Kaunas Cardiovascular Risk Cohort study started in 1977 (n = 1082, age 12–13 years). In 2012, a total of 507 individuals (63.9% of eligible sample) participated in the 35-year follow-up survey. Health examination involved measurements of blood pressure (BP), anthropometric parameters, and interview about health behaviours. Subjects were genotyped for *AGT* (M235T), *ACE* (I/D, rs4340), *ADM* (rs7129220), and *CACNB2* (rs12258967) genes polymorphisms. A genetic risk score was calculated as the sum of the number of risk alleles at each of four single nucleotide polymorphisms.

**Results:**

*AGT* TT genotype male carriers had the highest mean values of systolic BP in childhood. In females, *ADM* genotype AA was associated with the highest values of systolic and diastolic BP, while *CACNB2* genotype CC carriers had the highest values of diastolic BP in childhood. Systolic and diastolic BP in childhood, gain in BMI from childhood to adulthood, and risky alcohol consumption predicted hypertension in middle-aged men. In women, genetic risk score together with diastolic BP in childhood and gain in BMI were significant predictors of adult hypertension. The comparison of four nested logistic regression models showed that the prediction of hypertension improved significantly after the addition of BMI gain. Genetic risk score had a relatively weak effect on the improvement of the model performance in women.

**Conclusions:**

BP in childhood and the gain in BMI from childhood to adulthood were significant predictors of adult hypertension in both genders. Genetic risk score in women and risky alcohol consumption in men were independently related with the risk of adult hypertension.

## Introduction

Hypertension is one of the major cardiovascular risk factors which accounts for a large proportion of cardiovascular mortality, the main cause of death in Lithuania [1], [2]. High blood pressure (BP) affects approximately 40% of the adult population worldwide [3]. In Lithuania, prevalence of hypertension is very high: approximately 60% of men and 45% of women aged 25–64 have elevated BP [4]. Appropriate lifestyle modification can delay or prevent hypertension [5]. Preventive interventions are more likely to be successful when aimed at individuals who have a higher risk for developing hypertension. Identification of high-risk individuals earlier in life course might help to increase the effect of preventive measures.

There is extensive evidence that the roots of adult hypertension go back to childhood. A large number of longitudinal studies demonstrated different degrees of BP tracking from childhood to adulthood [6]. Moreover, cohort studies revealed the association of BP in early adulthood with childhood overweight and obesity, family history of hypertension, and low parental occupational status [7]–[9]. The data also showed that the increase in body mass index (BMI) from childhood to adulthood was positively related to the risk of adult hypertension [10]. Furthermore, the role of behavioural risk factors in the development of hypertension was widely investigated. High sodium diet, low intake of fruits and vegetables, risky alcohol consumption, and low physical activity were linked to higher risk of hypertension [5], [11]–[13].

In addition to environmental factors, a number of single nucleotide polymorphisms (SNPs) were shown to be linked to BP [14], [15]. Genes associated with renin-angiotensin-aldosterone system, such as angiotensinogen (*AGT*), angiotensin converting enzyme (*ACE*) and angiotensin II receptor 1 (*AGTR1*), were the most extensively studied as hypertension candidate genes [16]–[18]. A recent analysis from genome-wide association studies identified novel genetic variants associated with BP and hypertension [15], [19], [20]. Findings of the Cardiovascular Risk in Young Finns Study indicated that prediction of adult hypertension was enhanced when genetic risk score was taken into account [7].

In Lithuania, twenty years follow-up data of the Kaunas Cardiovascular Risk Cohort study demonstrated that childhood BP and gain in BMI were the best predictors of BP in early adulthood [21]. To date, the contribution of childhood BP and BMI to the risk of hypertension in middle-aged population has not been clearly established. In previous follow-up study, the association between behavioural factors and the risk of hypertension has not been examined. Finally, the role of genetic factors in prediction of hypertension has never been studied in Lithuania.

The aim of this study was to examine the independent effects of physical, behavioural and genetic factors identified in childhood and mid-adulthood for prediction of adult hypertension.

## Materials and Methods

### Ethics Statement

The study protocol was approved by the Lithuanian Bioethics Committee. Written consent on behalf of the children enrolled in the first survey (1977) was obtained from parents or guardians. Written informed consent for the participation in the follow-up survey (2012) was obtained from all participants.

### Study Design and Sample

The Kaunas Cardiovascular Risk Cohort study started in 1977 within the framework of the International Study of Juvenile Hypertension [22]. A random sample of Kaunas schoolchildren born in 1964 (n = 1082) was examined in the first cross-sectional survey. Over 35 years, four follow-up examinations of this cohort have been conducted [21], [23]. The data of the first survey and the last follow-up survey carried-out in 2012 were analysed for the present study. Over the period of observation, 91 individuals (8.4%) died, 103 subjects (9.5%) emigrated from Lithuania, 4 (0.4%) individuals were seriously ill, and the addresses of 90 (8.3%) subjects were not available in the National Population Register. The eligible sample consisted of 794 subjects who were invited for medical examination. A total of 507 subjects (63.9% of eligible sample), aged 48–49 years, participated in the last follow-up survey. Baseline characteristics were compared between those who participated and those who did not participate in 2012 follow-up survey. No statistically significant differences of analysed childhood variables were found between the groups.

### Measurements

BP was measured from the right brachial artery with a standard mercury sphygmomanometer in the sitting position after 5 minutes of rest. BP measurements were performed to the nearest 2 mmHg. The first Korotkoff phase was used to determine systolic BP, and the fifth phase was used to determine diastolic BP. Three consecutive BP measurements were taken. The average of these three measurements was used in the analysis.

Hypertension in mid-adulthood was defined as systolic BP≥140 mmHg and/or diastolic BP≥90 mmHg or BP<140/90 mmHg using antihypertensive medication for the last two weeks before examination. Resting heart rate was recorded as beats per minute.

The height of participants, without shoes, was measured to the nearest centimeter with a stadiometer. The body weight of participants, wearing light indoor clothing and no shoes, was measured to the nearest 0.1 kg with standardised medical scales. BMI was calculated as weight divided by height squared (kg/m^2^). In childhood, overweight and obesity were defined using age and sex specific cut-off points for BMI recommended by the International Obesity Task Force [24]. Overweight in adulthood was defined as BMI of 25–29.9 kg/m^2^, and obesity as BMI equal to or higher than 30 kg/m^2^.

To examine the sodium intake, 24-hour recall method was used. A standard questionnaire was applied to obtain data on alcohol consumption. The questionnaire contained questions about a type and frequency of alcohol consumption and amount of alcohol consumed at one occasion. The amount of alcohol consumed at one occasion was recalculated into standard alcohol units (SAUs) using the following formula: SAUs  =  amount (in litres) × strength of alcoholic drink (beer −5%, wine −12%, strong alcohol −40%). One SAU equals to 10 g of ethanol. Then the amount of SAUs consumed during a month was calculated. Risky alcohol consumption was considered as drinking of 56 SAUs or more per month for men and drinking of 28 SAUs or more per month for women. Physical activity was assessed using the International Physical Activity Questionnaire (IPAQ) [25]. Physical activity of less than 600 MET-min/week was considered as low.

### Genetic Analysis

Genetic analysis was performed in 2012 survey. For DNA extraction, blood samples were collected from each individual in ethylenediaminetetraacetic (EDTA) tubes. DNA was extracted from peripheral blood leukocytes using a reagent kit- Sorpoclean Genomic DNA Extraction Module kit (Sorpo Diagnostics, Vilnius, Lithuania) according to manufacturer's instructions. Aliquots of purified DNA were stored at −20°C until use in real time-PCR analysis. Subjects were genotyped for *AGT* (M235T, rs699), adrenomedullin *(ADM)* (rs7129220), and beta-2 subunit of voltage-gated calcium channel *(CACNB2)* (rs12258967) genes polymorphisms with TaqMan allelic discrimination Assay-By-Design genotyping kits according to Applied Biosystems recommendations: C_1985481_20, C_30872739_10, C_31302374_10, respectively (Applied Biosystems, Foster City, CA, USA). Cycling conditions were preceded by a denaturing step at 95°C for 10 min, followed by 40 cycles of denaturation at 95°C for 30 s, annealing at 60°C for 1 min. Allele-specific fluorescence was then analysed on an ABI 7900HT Sequence Detection System with SDS v 2.1 (Applied Biosystems, Foster City, CA, USA).

To analyse *ACE* (ID, rs4340) polymorphism genomic DNA were amplified by PCR reaction. The flanking oligonucleotide primer pairs used to characterize the I/D polymorphism and produce a 700-bp fragment, corresponding to the I allele, and a 400-bp fragment, corresponding to the D allele were as follows: ′-AGGAGAGGAGAGAGACTCAAGCACG-3′ and 5′-GGCAGCCTGGTTGATGAGTTCC-3′ (TibMolBiol, Berlin, Germany). D/D homozygotes were amplified again with an insertion specific primer pair. A 335-bp PCR product only in the presence of the I allele was amplified using 5‘-TGGGACCACAGCGCCCGCCACTAC-3‘ as sense primer and 5‘-TCGCCAGCCCTCCCATGCCCATAA-3‘ as antisense primer [26].

Quality control for genotyping of *AGT*, *ACE, ADM*, and *CACNB2* genes was performed using the DNA sequencing.

A genetic risk score was calculated as the unweighted sum of the number of risk alleles (0, 1 or 2) at each of four SNPs.

### Statistical Analysis

Categorical variables were expressed as percentages and tested by the χ^2^ test. Hardy-Weinberg equilibrium was also assessed using the χ^2^ test. The normality of distribution of continuous variables was tested by Kolmogorov-Smirnov test. Means and standard deviations (SD) were presented for the normally distributed continuous variables while median and interquartile range was calculated for the distributions that did not meet the criteria of normality. Student t test was used to compare the mean values of normally distributed variables and Mann-Whitney test was applied for the comparison of non-normal distributions.

Logistic regression analysis was performed to build nested prediction models of adult hypertension. To avoid multicollinearity problems, childhood systolic BP and diastolic BP were included separately in the logistic regression models. The models sequentially included childhood BP (model 1), genetic risk score (model 2), change in BMI from childhood to adulthood (model 3), and risky alcohol consumption in adulthood (model 4). The analysis was performed separately for men and women. Childhood BP, genetic risk score, and change in BMI were treated as continuous variables. The models calibration was assessed with Hosmer – Lemeshow χ^2^ test. Lower χ^2^ values and higher P values in the test reflect better calibration. The additional prognostic information of extended models was assessed by testing the improvement in the log-likelihood ratio (likelihood ratio test - LRT). Lower P values indicated larger difference between predictive quality of the models. The ability of the models to predict adult hypertension was estimated with C statistic by calculating the area under the receiver-operating characteristic curve and the net reclassification improvement index (NRI) [27]. NRI was calculated to determine the extent to which incorporation of additional variables into the prediction models improve the reclassification of individuals to either higher (for cases) or lower (for not cases) adult hypertension risk categories. All statistical analyses were performed using statistical software package SPSS version 20.0 for Windows.

## Results

Baseline (1977) and follow-up (2012) characteristics of the study subjects are presented in Table 1. In the first survey, girls had higher systolic BP, BMI and heart rate compared with boys. Over 35 years of follow-up, systolic and diastolic BP increased more for men than for women resulting in the higher prevalence of adult hypertension among men (60.2%) compared with women (30.8%). BMI gain from childhood to adulthood was also greater in men. In childhood, every tenth boy and girl were overweight or obese, while in adulthood the prevalence of overweight and obesity reached 69.1% in men and 56.1% in women. In 2012 survey, sodium intake and the prevalence of risky alcohol consumption was higher in men than in women. Genetic risk score was similar in both genders.

**Table 1:** Characteristics of the study population in childhood and adulthood.

Characteristic	Men	Women	P value
	n = 231	n = 276	
**Childhood (12–13 years)**			
Systolic BP, mm Hg*	111.9 (10.6)	115.9 (12.1)	<0.001
Diastolic BP, mm Hg*	54.6 (9.9)	55.5 (10.9)	0.371
BMI, kg/m^2^**	17.8 (3.4)	18.6 (3.3)	0.003
Overweight prevalence, %	11.3	12.7	0.623
Heart rate (beats per min)*	82.7 (11.4)	88.3 (13.1)	<0.001
**Adulthood (48–49 years)**			
Systolic BP, mm Hg**	135.0 (24.0)	125.3 (21.8)	<0.001
Diastolic BP, mm Hg**	90.0 (13.5)	80.7 (12.3)	<0.001
Hypertension, %	60.2	30.8	<0.001
Heart rate (beats per min)*	71.1(11.5)	72.1(11.5)	0.308
BMI, kg/m^2^**	26.9 (5.6)	25.6 (6.6)	0.008
Obesity prevalence, %	25.2	21.4	0.341
Change in BMI from childhood to adulthood, kg/m^2^ *	9.1 (4.6)	7.6 (4.4)	<0.001
Intake of sodium, g/d**	3.9 (2.6)	2.5 (1.6)	<0.001
Low physical activity prevalence, %	25.5	26.4	0.724
Risky alcohol consumption prevalence, %	22.9	5.8	<0.001
Genetic risk score **	3 (2)	3 (1)	0.7

*Mean and standard deviation; **median and interquartile range.

Abbreviations: BP –blood pressure; BMI – body mass index.

The distribution of all analysed genotypes was according to the Hardy-Weinberg equilibrium. No significant differences in the frequencies of the *AGT, ADM, CACNB2* genotypes or alleles between men and women were found (Table 2). The distribution of *ACE* genotypes slightly differed between men and women (P = 0.048).

**Table 2:** Prevalence (%) of the *AGT, ACE, ADM, CACNB2* genotypes and allele frequency in the study population.

Genotypes	Men	Women
*AGT* genotypes, n, %		
MM	61 (26.4)	78 (28.3)
MT	112 (48.5)	125 (45.3)
TT	58 (25.1)	73 (26.4)
T allele frequency	0.49	0.49
*ACE* genotypes*, n, %		
II	71 (30.7)	77 (27.9)
ID	104 (45.0)	152 (55.1)
DD	56 (24.2)	47 (17.0)
D allele frequency	0.47	0.45
*ADM* genotypes, n, %		
AA	4 (1.7)	6 (2.2)
AG	47 (20.3)	56 (20.3)
GG	180 (77.9)	214 (77.5)
G allele frequency	0.88	0.88
*CACNB2* genotypes, n, %		
CC	95 (41.1)	121 (43.8)
CG	115 (49.8)	122 (44.2)
GG	21 (9.1)	33 (12.0)
G allele frequency	0.34	0.34

*P = 0.048 between men and women, using χ^2^ test.

Abbreviations: AGT – angiotensinogen; ACE - angiotensin converting enzyme; ADM - adrenomedullin; CACNB2 - beta-2 subunit of voltage-gated calcium channel.

Childhood systolic BP and diastolic BP were associated with body weight (Table 3). Overweight children had higher mean values of BP compared to children with normal weight. Systolic BP in boys differed according to *AGT* genotype. Namely, *AGT* genotype TT carriers had the highest mean values of systolic BP. In girls, carriers of *ADM* genotype AA had the highest values of systolic BP and diastolic BP. Diastolic BP was higher in girls with *CACNB2* genotype CC compared to girls with *CACNB2* genotype GG.

**Table 3:** Means (SD) of systolic and diastolic blood pressure of 12–13 years old boys and girls according to body weight and genotypes.

Body weight/genotypes	Systolic BP (mm Hg)	Diastolic BP (mm Hg)
**Boys**		
Body weight		
Normal	110.5 (9.8)*	54.1 (9.7)*
Overweight	122.3 (11.6)*	59.0 (10.2)*
*AGT*, M235T genotypes		
MM	109.7 (10.6)*	55.1 (8.2)
MT	111.7 (9.1)	53.8 (10.7)
TT	114.3 (12.9)*	55.8 (9.8)
*ACE*, genotypes		
II	112.2 (10.3)	55.1 (9.8)
ID	111.1 (11.2)	53.3 (10.2)
DD	112.8 (10.1)	56.6 (9.0)
*ADM* genotypes		
AA	106.3 (8.1)	54.3 (4.1)
AG	112.4 (10.3)	54.5 (9.5)
GG	11.8 (10.8)	54.7 (10.1)
*CACNB2* genotypes		
CC	111.6 (11.3)	53.7 (9.9)
CG	112.7 (10.0)	55.6 (10.1)
GG	108.4 (10.8)	53.7 (8.6)
**Girls**		
Body weight		
Normal	115.3 (12.0)*	54.9 (10.6)*
Overweight	119.8 (12.6)*	59.6 (11.8)*
*AGT*, M235T genotypes		
MM	116.4 (12.4)	55.7 (10.4)
MT	115.3 (11.8)	54.3 (10.4)
TT	116.6 (12.5)	57.3 (12.1)
*ACE*, genotypes		
II	116.9 (13.0)	53.4 (10.7)
ID	114.7 (11.7)	56.0 (10.9)
DD	118.3 (11.6)	57.2 (10.7)
*ADM* genotypes		
AA	127.3 (4.8)*	65.3 (5.4)*
AG	116.7 (10.8)	54.2 (10.)*
GG	115.4 (12.5)*	55.5 (10.9)
*CACNB2* genotypes		
CC	116.5 (11.5)	56.8 (11.5)*
CG	115.9 (12.9)	55.2 (10.4)
GG	113.9 (11.4)	51.8 (9.2)*

*Statistically significant difference using Student t test or analysis of variance with Bonfferoni correction.

Abbreviations: SD – standard deviation; BP – blood pressure; AGT – angiotensinogen; ACE - angiotensin converting enzyme; ADM - adrenomedullin; CACNB2 - beta-2 subunit of voltage-gated calcium channel.

Characteristics of hypertensive and non-hypertensive adults are provided in the table 4. In childhood, both hypertensive men and women had higher systolic BP than non-hypertensive individuals, while childhood diastolic BP was higher only in hypertensive women. Genetic risk score also differed between hypertensive and non-hypertensive women; however, no difference was found in men. BMI gain was higher in hypertensive individuals compared to the non-hypertensive participants. Excessive weight gain resulted in higher BMI in adults with raised BP compared to those with normal BP. Risky alcohol consumption was more common only among hypertensive men. Level of physical activity and sodium intake was similar between hypertensive and non-hypertensive individuals.

**Table 4:** Characteristics of hypertensive and non-hypertensive adults.

Variable	Hypertensive	Non-hypertensive	P value
**Men**			
Childhood systolic BP*	113.3 (10.8)	109.6 (10.0)	0.009
Childhood diastolic BP*	55.3 (10.1)	53.7 (9.4)	0.255
Childhood heart rate*	82.9 (11.0)	82.4 (12.1)	0.741
Childhood BMI, kg/m^2^**	17.9 (3.3)	17.7 (3.5)	0.941
Adulthood BMI, kg/m^2^**	28.3 (6.9)	25.4 (5.1)	<0.001
Change in BMI, kg/m^2^ *	10.5 (4.7)	7.2 (3.5)	<0.001
Genetic risk score **	4 (1)	3 (2)	0.15
Risky alcohol consumption prevalence, %	27.3	16.3	0.05
Low physical activity prevalence, %	24.5	27.2	0.14
Intake of sodium, g/d**	3.9 (2.4)	4 (2.8)	0.205
**Women**			
Childhood systolic BP*	118.3 (13.8)	114 (11.1)	0.033
Childhood diastolic BP*	59.5 (11.6)	53.7 (10.1)	<0.001
Childhood heart rate	89.2 (12.7)	87.9 (13.2)	0.452
Childhood BMI, kg/m^2^**	18.9 (4.4)	18.6 (3)	0.511
Adulthood BMI, kg/m^2^**	28.8 (9.6)	24.9 (5.7)	<0.001
Change in BMI, kg/m^2^ *	10.5 (4.8)	6.3 (3.5)	<0.001
Genetic risk score**	4 (2)	3 (2)	<0.001
Risky alcohol consumption prevalence, %	4.7	6.2	0.61
Low physical activity prevalence, %	24.4	26.6	0.227
Intake of sodium, g/d**	2.5 (1.7)	2.6 (1.6)	0.293

*Mean and standard deviation; ** median and interquartile range.

Abbreviations: BP – blood pressure; BMI – body mass index.

A logistic regression analysis was performed including variables that were associated with adult hypertension from univariate analysis. The significant predictors of adult hypertension in men were childhood systolic and diastolic BP (in separate models), BMI gain from childhood to adulthood and risky alcohol consumption (Tables 5 and 6). In women, childhood diastolic BP, genetic risk score and BMI gain were significantly associated with risk of hypertension. In models 1 and 2, childhood systolic BP significantly predicted hypertension in women; however, addition of the change in BMI to model 3 diminished the predictive value of systolic BP. Full model with childhood systolic BP explained 27.7% of the variation in adult hypertension for men and 31.3% of variation for women as estimated by Nagelgerke pseudo-R^2^ values. When childhood diastolic BP was used instead of systolic BP in the final model, Nagelgerke pseudo-R^2^ values were 24.6% and 36.2% for men and women, respectively.

**Table 5:** Comparison of models with childhood systolic blood pressure for the prediction of adult hypertension.

Variable	Model 1	Model 2	Model 3	Model 4
	OR (95% CI)	OR (95% CI)	OR (95% CI)	OR (95% CI)
**Men**				
Systolic BP*	**1.45**	**1.43**	**1.65**	**1.67**
	**(1.09–1.91)**	**(1.08–1.89)**	**(1.21–2.25)**	**(1.21–2.29)**
Genetic risk score		1.17	1.08	1.06
		(0.91–1.36)	(0.87–1.33)	(0.86–1.32)
Change in BMI,			**1.25**	**1.26**
kg/m^2^			**(1.15–1.36)**	**(1.16–1.37)**
Risky alcohol				**2.46**
consumption				**(1.18–5.16)**
C statistic	0.588	0.604	0.766	0.777
(95% CI)	(0.513–0.663)	(0.530–0.679)	(0.704–0.828)	(0.715–0.838)
LRT (P)		0.28	<0.001	<0.014
NRI (P)**		0.101 (0.15)	0.475 (<0.001)	0.477 (<0.001)
Hosmer-Lemeshow, χ^2^ (P)	3.97 (0.86)	8.98 (0.34)	4.37 (0.82)	7.49 (0.49)
**Women**				
Systolic BP*	**1.32**	**1.29**	1.30	1.30
	**(1.02–1.71)**	**(1.01–1.68)**	(0.97–1.73)	(0.97–1.73)
Genetic risk score		**1.37**	**1.38**	**1.37**
		**(1.12–1.68)**	**(1.10–1.73)**	**(1.09–1.72)**
Change in BMI,			**1.29**	**1.29**
kg/m^2^			**(1.19–1.39)**	**(1.19–1.39)**
Risky alcohol				0.70
consumption				(0.17–2.99)
C statistic	0.551	0.628	0.789	0.788
(95% CI)	(0.473–0.628)	(0.557–0.698)	(0.731–0.847)	(0.730–0.847)
LRT (P)		0.02	<0.001	0.628
NRI (P)**		0.271 (0.009)	0.587(<0.001)	0.587(<0.001)
Hosmer-Lemeshow, χ^2^ (P)	15.37 (0.05)	6.34 (0.61)	4.29 (0.83)	5.37 (0.72)

*OR for a 1-SD increase in systolic BP; **Model 1 was used for comparison.

Abbreviations: OR – odds ratio, CI – confidence interval; BP – blood pressure; BMI – body mass index; LRT - likelihood ratio test; NRI - net reclassification improvement index.

**Table 6:** Comparison of models with childhood diastolic blood pressure for the prediction of adult hypertension.

Variable	Model 1	Model 2	Model 3	Model 4
	OR (95% CI)	OR (95% CI)	OR (95% CI)	OR (95% CI)
**Men**				
Diastolic BP*	1.17	1.17	**1.36**	**1.38**
	(0.89–1.53)	(0.89–1.52)	**(1.01–1.85)**	**(1.01–1.88)**
Genetic risk score		1.14	1.10	1.09
		(0.34–1.39)	(0.89–1.36)	(0.88–1.35)
Change in BMI,			**1.25**	**1.26**
kg/m^2^			**(1.15–1.36)**	**(1.16–1.36)**
Risky alcohol				**2.41**
consumption				**(1.17–1.97)**
C statistic	0.551	0.561	0.750	0.759
(95% CI)	(0.476–0.627)	(0.485–0.637)	(0.687–0.813)	(0.697–0.822)
LRT (P)		0.212	<0.001	0.014
NRI (P)**		0.111 (0.285)	0.430 (<0.001)	0.774 (<0.001)
Hosmer-Lemeshow, χ^2^ (P)	8.97 (0.35)	1.66 (0.99)	6.98 (0.54)	4.17 (0.84)
**Women**				
Diastolic BP*	**1.75**	**1.65**	**1.89**	**1.89**
	**(1.32–2.31)**	**(1.25–2.18)**	**(1.36–2.63)**	**(1.36–2.64)**
Genetic risk score		**1.31**	**1.28**	**1.28**
		**(1.06–1.61)**	**(1.01–1.62)**	**(1.01–1.62)**
Change in BMI,			**1.31**	**1.31**
kg/m^2^			**(1.21–1.42)**	**(1.21–1.42)**
Risky alcohol				1.04
consumption				(0.22–4.97)
C statistic	0.649	0.673	0.814	0.814
(95% CI)	(0.578–0.721)	(0.603–0.743)	(0.759–0.868)	(0.759–0.868)
LRT (P)		0.01	<0.001	0.965
NRI (P)**		0.377 (<0.001)	0.500 (<0.001)	0.500 (<0.001)
Hosmer-Lemeshow, χ^2^ (P)	5.41 (0.71)	3.57 (0.89)	5.33 (0.72)	7.0 (0.54)

*OR for a 1-SD increase in diastolic BP; **Model 1 was used for comparison.

Abbreviations: OR – odds ratio, CI – confidence interval; BP – blood pressure; BMI – body mass index; LRT - likelihood ratio test; NRI - net reclassification improvement index.

The comparison of four nested models showed that the addition of risk variables had different effects on model performance in men and in women. According to the likelihood ratio tests, the addition of BMI gain and risky alcohol consumption improved the performance of prediction models in men (Table 5 and 6). When the change in BMI was included into model 3, C statistic increased significantly showing better discrimination of the model. Also, model 3 that included childhood BP, genetic risk score and change in BMI, demonstrated the highest net reclassification improvement compared to model 1. In women, the inclusion of genetic risk score into the model 2 improved the prediction power of both models with childhood systolic and diastolic BP (NRI 0.271 and 0.377, respectively). The greatest improvement in the model performance was observed when BMI gain was added into model 3. C statistic reached 0.789 in the model with systolic BP and 0.814 in the model with diastolic BP. The addition of BMI gain had a moderate effect on reclassification (NRI was 0.587 and 0.500 in the models with systolic BP and diastolic BP, respectively).

## Discussion

The present study demonstrated that the predictors of adult hypertension differed for men and women. Namely, childhood BP, BMI gain from childhood to adulthood, and risky alcohol consumption predicted hypertension in middle-aged men. Study results suggest that inclusion of BMI gain significantly improves the performance of prediction model. In women, genetic risk score together with childhood diastolic BP and gain in BMI were significant predictors of adult hypertension. It should be noted that genetic risk score had a relatively weak effect on the improvement of the model performance. Similarly to men, prediction of hypertension in middle-aged women improved significantly after the addition of the change in BMI from childhood to adulthood.

Several prospective studies investigated the predictors of adult hypertension and found that childhood BP and overweight were associated with a higher risk of elevated BP in adulthood [7], [10], [28]. Our data confirmed the association between childhood BP and adult hypertension. Childhood BMI, however, was not related to adult hypertension although overweight children had higher values of BP than children with normal weight. In this study, BMI gain had the greatest effect on the risk of adult hypertension, partly because excessive gain was associated with higher BMI in adulthood. The pooled analysis of three British birth cohorts also found that exposure to overweight in early life did not influence the adult BP, whereas obesity in adulthood accounted predominantly for the risk of hypertension [29]. The systematic review on childhood obesity and adult cardiovascular disease risk concluded that the risk of raised BP was highest in individuals who had low BMI in childhood and became overweight in adulthood [30]. On the other hand, the joined analysis in four longitudinal cohort studies showed that those overweight children who became non-obese adults had a similar risk of adult hypertension as never obese individuals [28]. In Kaunas cohort, only few overweight children had normal BMI in adulthood. Over 35 years of follow-up, the prevalence of overweight and obesity increased dramatically, i.e. by 6.1 times in men and by 4.4 times in women. Excessive gain in BMI resulted in a very high prevalence of adult hypertension. Namely, almost two thirds of men and one third of women being 48–49 years old had an increased BP. From a public health perspective, interventions during childhood may be important in prevention of adult obesity. Promotion of healthy nutrition and physical activity earlier in the life course might help to avoid excessive weight gain and to prevent high BP and metabolic complications.

In addition to overweight, alcohol consumption increased the risk of hypertension in Kaunas men. Epidemiological studies have established a close relationship between alcohol consumption and BP [12], [31]. Higher levels of consumption of all types of alcoholic beverages were associated with a higher risk of hypertension. The effect of alcohol on BP was most closely related to the current pattern of consumption [32]. A biphasic effect of alcohol on BP (the acute vasodilator effect of ethanol with a long-term increase in BP) was observed by some authors [32], [33]. High alcohol consumption is a serious problem in Lithuania. The data of Lithuanian health behaviour monitoring among population aged 20–64 showed that alcohol consumption had increased over the last two decades, especially among women [34]. In 2012, two thirds of men and one fifth of women reported regular drinking of any alcohol at least once a week [35]. This is a matter of public health concern, because alcohol consumption causes a lot of social and health problems in the country including acute effect of alcohol on the risk of death from cardiovascular diseases [36].

There is strong evidence that high consumption of salt is the major factor responsible for increase in BP [5]. In our study, the mean intake of sodium per day in men (3.9 g) as well as in women (2.5 g) was higher than recommended for the prevention of hypertension (2 g sodium/day) [37]. High consumption of salt was typical for the majority of the study population; therefore, the possibility to evaluate the effect of low salt intake on BP level with sufficient power was limited. This could partially explain why sodium intake was not related to adult BP in our study.

Epidemiological studies suggest that regular aerobic physical activity may be beneficial for prevention and treatment of hypertension [13], [38]. Larger improvements in BP levels may be gained from higher activity and fitness levels. The Coronary Artery Risk Development in Young Adults (CARDIA) study revealed that the physical activity that did not raise fitness levels might not lower the risk of hypertension [13]. In Kaunas cohort, there was no difference in physical activity level between hypertensive and non-hypertensive individuals. The level of physical activity was defined using IPAQ which includes not only moderate and high intensity exercises, but also walking. Moreover, walking accounted for a substantial part of METs (data are not shown). Possibly, walking did not have sufficient impact on physical fitness and risk of hypertension.

In women, the prediction of hypertension improved when genetic risk score was added to the model. For evaluation of genotype effect on the risk of hypertension, four SNPs were chosen: two SNPs (*AGT* and *ACE*) related to renin-angiotensin-aldosterone system and other two SNPs (*ADM* and *CACNB2*) newly identified in genome-wide association studies. The renin-angiotensin-aldosterone system plays a key role in BP regulation. *AGT* gene encoding angiotensinogen and *ACE* gene encoding angiotensin-converting enzyme were investigated most extensively and have been shown to be associated with hypertension [16]–[18]. The 235T allele of the *AGT* gene is linked to an increased level of circulating angiotensinogen [39]. The level of circulating ACE depends on *ACE* gene insertion/deletion (I/D) polymorphism [40]. Meta-analyses of genome-wide association studies have identified regions of the genome that are significantly associated with BP control [15], [19], [20]. Among new identified loci for hypertension there are genes encoding ADM and CACNB2. ADM is a biologically active hypotensive peptide with multifunctional properties to control circulation and body fluid volume regulation. It is a potent vasodilator and natriuretic peptide [41]. *CACNB2* gene encodes the beta-2 subunit of a voltage-gated calcium channel, which plays an essential role in regulating the surface expression and gating properties of the channels. Opening of the voltage-gated calcium channels enables a transmembraneous influx of calcium ions [42].

The effect direction of all analysed SNPs on BP was similar in Kaunas Cardiovascular Risk study compared with the data of other studies; however, the power of the study was too low to show significant associations for individual SNPs. Several significant associations between genes and BP were found only in childhood. Potential interactions between genetic and environmental determinants of BP, such as body mass, diet, physical activity, alcohol consumption, influence BP level during life course and can possibly explain the lower impact of individual SNPs on adult hypertension. Genetic risk score aggregates information from multiple risk SNPs being more effective than individual SNPs for measurement of disease risk.

In this study, genetic risk score was associated with an increased risk of hypertension in women but not in men. Some other studies have also shown gender differences in the effect of genes on BP [39], [43]. In Copenhagen City Heart study, *AGT* genotype TT was associated with an increased risk of hypertension only in women [39]. An association between *ACE* genotype DD and increased level of diastolic BP was found in men, but not in women in the Framingham Heart Study [43]. Gender-specific associations could be plausible, because the estrogen-related factors may mediate the genetic predisposition to elevated BP.

The strength of the present study includes use of the data from a randomly selected cohort prospectively followed up for 35 years. The environmental, genetic and behavioural determinants of hypertension were carefully measured in childhood and mid-adulthood. BP was measured using the same methodology in the first and in the last survey.

The study has a few limitations. During the long term follow-up, the loss of participants was quite substantial. One of the reasons for this was a high rate of emigration from Lithuania over the last decades. Although no differences between participants and non-participants were found in baseline measurements, the influence of environmental factors on BP level during the life course might be different in those groups. In addition, genetic analysis was performed only in the last survey; therefore, genotype data were not available for non-participants. The loss to follow-up might have resulted in selection bias if non-response was associated with genetic risk score. Furthermore the study sample was relatively small, affecting its ability and power to detect the effect of genetic factors on the risk of hypertension. Finally, only few SNPs from the long list of genes possibly associated with BP were analysed.

### Conclusions

Childhood BP and the gain in BMI from childhood to adulthood were significant predictors of adult hypertension in both genders. In women, genetic risk score was independently related to the risk of hypertension; however, genetic risk score had a relatively weak effect on the improvement of the predictive model performance. In men, prediction of adult hypertension was improved after the addition of risky alcohol consumption into the model. The results of this study have relevant public health implications demonstrating importance of identification of children who have high risk of hypertension in adulthood and emphasising the need of health promotion activities throughout the life course.
